# An overlooked connection: serotonergic mediation of estrogen-related physiology and pathology

**DOI:** 10.1186/1472-6874-5-12

**Published:** 2005-12-20

**Authors:** Leszek A Rybaczyk, Meredith J Bashaw, Dorothy R Pathak, Scott M Moody, Roger M Gilders, Donald L Holzschu

**Affiliations:** 1 Integrated Biomedical Science Graduate Program, The Ohio State University, 1190 Graves Hall, 333 West 10th Avenue, Columbus, OH, 43210-1218, USA; 2 Department of Psychology, 200 Porter Hall, Ohio University, Athens, OH 45701, USA; 3 Departments of Epidemiology and Family Practice, A641 West Fee Hall, Michigan State University, East Lansing, MI48824, USA; 4 Department of Biological Sciences, 318 Irvine Hall, Ohio University, Athens, OH 45701-2939, USA; 5 School of Recreation and Sport Sciences, E184 Grover Center, Ohio University, Athens, Ohio 45701, USA; 6 Department of Biological Sciences, 239 Life Sciences Building, Ohio University, Athens, OH 45701, USA

## Abstract

**Background:**

In humans, serotonin has typically been investigated as a neurotransmitter. However, serotonin also functions as a hormone across animal phyla, including those lacking an organized central nervous system. This hormonal action allows serotonin to have physiological consequences in systems outside the central nervous system. Fluctuations in estrogen levels over the lifespan and during ovarian cycles cause predictable changes in serotonin systems in female mammals.

**Discussion:**

We hypothesize that some of the physiological effects attributed to estrogen may be a consequence of estrogen-related changes in serotonin efficacy and receptor distribution. Here, we integrate data from endocrinology, molecular biology, neuroscience, and epidemiology to propose that serotonin may mediate the effects of estrogen. In the central nervous system, estrogen influences pain transmission, headache, dizziness, nausea, and depression, all of which are known to be a consequence of serotonergic signaling. Outside of the central nervous system, estrogen produces changes in bone density, vascular function, and immune cell self-recognition and activation that are consistent with serotonin's effects. For breast cancer risk, our hypothesis predicts heretofore unexplained observations of the opposing effects of obesity pre- and post-menopause and the increase following treatment with hormone replacement therapy using medroxyprogesterone.

**Summary:**

Serotonergic mediation of estrogen has important clinical implications and warrants further evaluation.

## Background

In mammalian females, estrogen that acts extracellularly is primarily produced in the reproductive organs, and concentrations in blood serum and other tissues change over the lifespan and within the ovarian cycle[1]. The most active and most studied form of estrogen in mammals is 17-β estradiol (hereafter E2), although less active forms are also present [2]. Changes in E2 typically occur in conjunction with changes in progesterone, and are to some degree dependent on progesterone priming. In this paper, we will primarily focus on physiological levels of E2 assuming the presence of progesterone between puberty and menopause, and assuming its absence after menopause. Differences in estrogen concentrations are associated with physiological changes affecting the central nervous system (CNS), skeletal, vascular, and immune systems. The mechanisms producing some of these changes have yet to be fully elucidated [3].

Estrogen receptors and serotonin receptors coexist in cells in a wide variety of tissues, and this critical review of the literature suggests that many of E2's effects may be mediated by changes in the actions of serotonin (5HT). Serotonin is usually considered to be a neurotransmitter, but surprisingly, only 1% of serotonin in the human body is found in the CNS [4]. The remaining 99% is found in other tissues, primarily plasma, the gastro-intestinal tract, and immune tissues, where serotonin acts as a hormone regulating various physiological functions including vasodilation[5], clotting[6], recruitment of immune cells [7-9], gastro-intestinal motility,[10] and initiation of uterine contraction [11,12]. Serotonin also has peripheral functions in a wide variety of animal phyla [13-16] and is similar in chemical structure to auxin, which regulates plant cell shape, growth, and movement [17].

Both naturally-occurring and pharmacologically-induced changes in E2 alter the concentration of serotonin through two mechanisms. First, E2 increases production of tryptophan hydroxylase[18,19] (TPH, the rate-limiting step in synthesis of serotonin from tryptophan), increasing the concentrations of serotonin in the body [20,21]. Second, E2 inhibits the expression of the gene for the serotonin reuptake transporter (SERT) and acts as an antagonist at the SERT, thus promoting the actions of serotonin by increasing the time that it remains available in synapses and interstitial spaces [22,23].

Beyond increasing concentrations of serotonin, E2 also modulates the actions of serotonin because the activation of E2 receptors affects the distribution and state of serotonin receptors. Higher levels of E2 in the presence of progesterone upregulate E2 β receptors (ERβ) and down regulate E2 α receptors (ERα) [24]. ERβ activation results in upregulation of the 5HT_2A _receptor,[25] while ERα activation results in an increase in 5HT_1A _receptors via nuclear factor kappa B (NFkB) [26]. Therefore, increasing E2 causes an increase in the density and binding of the 5HT_2A _receptor,[27,28] which could explain the observed increases in 5HT_2A _density for post-menstrual teenage girls [29]. 5HT_2A _activity stimulates an increase in intracellular Ca^++^,[30] which causes changes in cellular function [17,31]. 5HT_2A _activation subsequently causes Protein Kinase C (PKC) activation. The effects of increased Ca^++ ^and PKC in cells are system-specific and explain many of the physiological consequences of serotonin activation. One effect of PKC activation is the uncoupling of 5HT_1A _auto-receptors[32] and decreasing serotonin's effect at these receptors [33,34]. Following 5HT_2A _activation of PKC, 5HT_1A _receptors become unable to reduce serotonin production through negative feedback, and serotonin concentrations increase [32-34] E2 compounds this effect by directly inhibiting 5HT_1A _function [35,36].

With reduced levels of E2, 5HT_1A _receptors are disinhibited and counter the effects of 5HT_2A _receptor activation. Increased activation of 5HT_1A _in the immune system results in greater mitotic potential via cyclic adenosine monophosphate (cAMP) and extra cellular response kinase (ERK) [37-40]. Additionally, the reinstatement of 5HT_1A _auto-regulation decreases serotonin concentrations by allowing negative feedback inhibition of serotonin production and release. Normal physiology depends on maintaining a balance between 5HT_2A _receptor produced Ca^++ ^inflow and 5HT_1A _receptor suppression of cAMP production. Pathologies result when this balance is perturbed, and the specific manifestation of these pathologies depend on which system is affected.

The current literature documents a wide range of individual effects of both estrogen and serotonin, which have been successfully used to explain both normal and pathological processes. E2, for example, initiates the development of the female reproductive system, influences the deposition of body fat, regulates the production of prolactin and other hormones, and increases sodium and water retention [41]. Independent of estrogen, serotonin regulates urination, influences the production of cerebrospinal fluid, and relaxes vascular smooth muscle [42]. These effects can be accounted for without reference to the interaction between E2 and serotonin. However, we hypothesize that considering how estrogen's actions might be mediated by serotonin explains findings that would not be predicted by either action alone and suggests possible treatment strategies that have not yet been considered. It is beyond the scope of this paper to provide an exhaustive catalog of the individual effects of either E2 or serotonin; we will limit our discussion to the physiological consequences of E2 that are consistent with the known functions of serotonin and its receptors.

## Discussion

### The central nervous system

Changes in estrogen are correlated with a variety of effects in the CNS, such as changes in pain transmission, headache, dizziness, nausea, temperature regulation, and mood [41]. Serotonin systems regulate these same functions[41,43] in a direction consistent with mediation of E2 effects. For pain, E2 acts as a central analgesic,[44] and pain sensation is inhibited by the activation of some serotonergic neurons [4]. Analgesic drugs that exploit this effect at the 5HT_2A _receptor are already available [4,45-48]. We hypothesize that E2's upregulation of the 5HT_2A _receptor in the brain might contribute to E2-mediated pain relief, in which case central administration of 5HT_2A _receptor antagonists would decrease E2's analgesic effects. In the spinal cord, altered expression of 5HT_2A _receptors can both increase and decrease pain [48,49]. E2's upregulation of 5HT_2A _in the spinal cord could be a factor in the development of fibromyalgia, which presents as increased generalized pain sensation. Serotonergic regulation of fibromyalgia is supported by evidence that fibromyalgia is comorbid with other serotonin-related pathologies,[50] and that fibromyalgia patients have altered tryptophan metabolism[51] and can be treated with 5HT_2A _antagonists [50]. E2's effect on serotonin could also explain why fibromyalgia is more frequently observed in females than males [52].

Females are also at greater risk for headaches,[43] which can result from vasodilation in the brain [53]. Activation of an additional serotonin receptor, 5HT_1B_, is one mechanism by which vasodilation occurs. 5HT_1B _receptors are not uncoupled by E2 (unlike 5HT_1A _receptors), and their vasodilatory effect is typically balanced by activation of 5HT_2A _receptors, which result in vasoconstriction [54]. After E2 exposure, increased serotonin concentrations result in greater activation of both the 5HT_1B _and 5HT_2A _receptors. Under normal conditions, upregulation and activation of 5HT_2A _receptors enable them to balance the effects of 5HT_1B _receptors [27,28,55]. We suggest that females' increased headache risk might result if high serotonin concentrations are maintained without adequate compensatory 5HT_2A _activity.

Two of the major side effects of E2 treatment are dizziness and nausea, which are controlled in the CNS. The mechanism by which these side effects occur has not been fully elucidated. It is possible that E2's effect on serotonin pathways is responsible for these symptoms, as 5HT_2A _receptors activate vestibular neurons (which maintain balance)[56] and are found in emetic centers, which are involved in chemically-induced vomiting [57]. Our hypothesis is corroborated by the use of serotonergic drugs to minimize these side effects of E2 treatment [58].

The loss of estrogen at menopause results in decreased density of 5HT_2A _receptors and lower activity of serotonin, which could explain aberrant temperature regulation, including hot flashes and night sweats. Although the effects of temperature changes are felt throughout the body, 5HT_2A _receptors in the CNS are responsible for temperature regulation. Administration of drugs acting at the 5HT_2A _receptor restores normal temperature regulation following ovariectomy[59] and chemically induced changes in body temperature[60] The nighttime prevalence of hot flashes and night sweats could be a result of the conversion of serotonin to melatonin at night, resulting in lower circulating serotonin levels [61]. Phytoestrogens preferentially bind to ERβ receptors[30] and are effective at reducing hot flashes and night sweats [62]. The mechanism by which these compounds work could be an ERβ-produced upregulation of 5HT_2A _receptors.

Depression is more common in women than in men and is known to be mediated by serotonin receptor levels [43,63]. Specifically, depression is linked to decreased density of serotonin receptors and decreased efficacy of serotonin in the brain. The increased risk, timing of onset, and effectiveness of treatment of depression in women may be mediated by estrogen's effect on serotonin receptors. The onset of depression in women is a characteristic of times when estrogen levels are relatively low (in early pregnancy, postpartum, and around and following menopause) or low in comparison to progesterone (the luteal phase of the menstrual cycle) [64,65]. In women with depression around or following menopause, the effectiveness of treatment with selective serotonin reuptake inhibitors (SSRIs) is enhanced by simultaneous administration of estrogen,[63] and doses of estrogen alone are effective at treating premenstrual, postpartum, and perimenopausal depression, especially for depression linked to aberrant expression of 5HT_2A _receptors [25,66]. ERβ regulates the antidepressant effect of E2 in mice; ERβ knockout mice fail to show the decrease in immobility usually induced by E2 doses in a forced swim test [67]. The increased levels of serotonin and increased activity of the 5HT_2A _receptor caused by E2 could be the mechanism for E2's antidepressant effects, in which case 5HT_2A _receptor agonists could also enhance the anti-depressant effects of E2.

### The skeletal system

Estrogen and serotonin also affect the skeletal system. As bones grow, they are continually remodeled and reshaped. Normal bone development is affected by growth hormone, parathyroid hormone, calcitonin, and environmental factors like dietary calcium intake and physical activity. In addition to these factors, estrogen and serotonin play an important role in the development and maintenance of bone mass. For bone growth to occur, two types of cells are required: osteoblasts, which form new bone, and osteoclasts, which resorb bone. During puberty, osteoclasts and osteoblasts are in balance and resorb and build bone simultaneously, but osteoporosis results when osteoclasts increase relative to osteoblasts. These effects have been linked to E2 concentrations in both males and females,[68,69] and we propose that they can be explained by examining E2-produced changes in serotonergic function in bone growth and loss. 5HT_2A _receptor activation causes an increase in expression of osteoblast progenitor cells, maintaining bone density [70]. SERT activation, in contrast, increases osteoclasts in bone, aiding in bone growth in childhood,[71] but resulting in loss of bone density and increases in extracellular Ca^++ ^postpartum[72,73] and in menopause [74,75]. Studies of female mice lacking the ERα, the ERβ, or both suggest these two receptors might counterbalance each other's effects on longitudinal bone growth,[76] with ERβ primarily responsible for decreasing bone growth and increasing bone resorption [77]. Because ERα and ERβ have opposing effects on serotonin systems, we hypothesize that mediation by serotonin could explain E2's effects on the skeletal system: the decrease in bone density observed following menopause or when E2 function is otherwise compromised. However, bone loss begins around age 30 in men and women and this early bone loss cannot be entirely explained by differences in E2 concentrations or by our proposed model [78].

### The vascular system

In the vascular system, estrogen and serotonin have been shown to individually alter clotting, cholesterol, vasoconstriction, and heart attacks. Both high and low levels of E2 have been associated with increased risk of thromboembolism; high levels result in increased clot formation, while low levels result in slower clot breakdown. Unusually high concentrations of estrogen (beyond normal physiological levels) directly increase the likelihood of clotting by increasing production of clotting factors VII through X in the liver [41]. In addition, these levels of E2 might increase clotting by increasing serotonin, which is constitutively present in human plasma and platelets and works to promote clotting[6,79] and increase density of platelets [58]. Increased clotting and thromboembolism at low concentrations of E2 [80] can also be explained using serotonergic changes. Postmenopausal women have longer latency to lysis of clots, and E2 replacement therapy returns latencies to pre-menopausal levels [81]. Patients with slower clot breakdowns have decreased uptake and release of serotonin from platelets,[82] and at low E2 levels serotonin's ability to break down clots via the 5HT_2A _receptor is limited,[83,84] so we suggest that lower serotonin activity associated with lower E2 levels could also contribute to increased clotting.

Increased concentrations of E2 are also associated with decreased cholesterol, and at menopause, there is an increase in total serum cholesterol, which is reduced by estrogen-containing hormone replacement therapy [85]. We suggest higher cholesterol after menopause is linked to the effects of serotonin. Serotonin increases membrane fluidity by incorporation of cholesterol into membranes, decreasing bioavailable cholesterol [86,87]. Increased membrane fluidity also increases serotonergic function, creating a positive feedback loop [88,89]. If serotonin is an intermediary between estrogen and cholesterol, then in the presence of high concentrations of E2, we would expect more cholesterol incorporated into membranes, thereby reducing cholesterol present in the plasma. Our hypothesis would be supported if the administration of drugs that reduce concentrations of serotonin in the plasma cause increases in plasma cholesterol despite consistent levels of E2.

Both clotting and cholesterol contribute to heart attack risk. Women are at lower risk of heart attack than men prior to menopause, but changes in the vascular system after menopause result in the loss of protection from heart disease [41,43]. In females, recent evidence suggests that physiological levels of E2 protect against heart attacks, while testosterone makes heart attacks more likely [90]. E2 acting at ERβ is responsible for this protective effect, as mice lacking ERβ have greater mortality and increased heart failure indicators following experimentally induced myocardial infarctions [91]. We hypothesize that these effects in females can be explained in part by serotonin receptor changes. Specifically, in the presence of physiological E2 and therefore ERβ activation, serotonin preferentially acts on 5HT_2A _receptors and to reduce vasospasm in cardiac tissue. After menopause, when 5HT_2A _receptors have been down regulated, serotonin instead acts on 5HT_1A _receptors, which cause adrenergic stimulation of smooth muscle[92] and increase likelihood of cardiac vasospasm [93]. This increases the risk of heart attack [92,94-96]. In addition, testosterone, which increases following menopause, compounds the actions of serotonin at 5HT_1A _receptors by preventing desensitization of 5HT_1A _receptors [97]. These changes in sensitivity of cardiac vessels, combined with increased clotting and lipid levels, would be expected to increase heart attack risk, arteriosclerosis and strokes. However, E2 is not solely responsible for protection from heart attack, progesterone also plays a role. Hormone replacement therapy (HRT) containing E2 and medroxyprogesterone instead of E2 and progesterone has been shown to increase heart attack [98]. Although the study showing increased heart attack risk during HRT is controversial,[99] it is possible that decreased concentrations of serotonin produced by treatment with medroxyprogesterone[93,100] could contribute to this increased risk.

### The immune system

Both E2 and serotonin are also active in the immune system, and in this system, their interaction is well-documented. E2 suppresses major histocompatibility complex II (MHC II) proteins in a tissue-specific manner [101] and acts centrally to suppress the immune system[102] by helping to activate 5HT_2A _receptors in the thymus [28,103-105]. Estrogen treatment also indirectly suppresses MHC II protein expression via serotonin [102,106]. Specifically, increased 5HT_2A _activity causes decreased MHC II production,[107] and decreased selection against self-reactive helper T cells (T_H_1) [108]. In addition, the concurrent inactivation of 5HT_1A _receptors decreases TNF-α production [109,110]. Although self-reactive T_H_1 cells are present, we hypothesize that E2's suppression of MHC II prevents them from becoming activated, and therefore while sufficient E2 is present they fail to attack tissues. Following menopause, or when E2 levels are unusually low, suppression of MHC II and immune function is lost, allowing self-reactive T_H_1 cells to become active and pathogenic. It is possible that estrogen and serotonin's modulation of the immune system prevents immune attack on offspring during pregnancy (when estrogen is at relatively high concentrations) and avoids infection after delivery (when estrogen is relatively low) [111].

MHC II protein and self-reactive T cells appear to be the common denominators among autoimmune disorders in women, suggesting a role for E2 and serotonin in mediating these disorders. Multiple sclerosis (MS) is associated with the presence of MHC II protein polymorphic pathogenic alleles[112,113] and serotonin depletion[114] This serotonin depletion could be a consequence of low E2, so the decrease in MS symptoms during pregnancy [115] could be explained by higher concentrations of E2. Also the severity of MS symptoms increases as serotonin levels decrease[116], symptoms worsen in phases of the menstrual cycle when there is low E2[117], and low levels of E2 result in changes in the 5HT signaling pathway [118]. In female SERT knockout mice, symptoms of experimental allergic encephalomyelitis (a MS model) are less severe and have a greater latency to occurrence, possibly as a result of increased serotonin availability [119]. Not only may low serotonin levels be linked to MS, but the effects of serotonin on MS may involve 5HT_2 _receptors in particular. Gene-microarray analysis of brain lesions found lower 5HT_2 _receptor expression in all 4 MS patients that analysis was preformed for compared to that of 2 controls [120].

Serotonin depletion could also be produced by conversion of serotonin to melatonin in the absence of light, which might explain the increased incidence of MS in more northern climates[121] (where daylight periods are shorter) and the reason that light therapy can be effective in reducing symptoms of MS [122]. Similarly, self-reported incidence of Type I diabetes (IDDM) is negatively correlated with exposure to UV radiation and positively correlated with latitude in Australia [123]. Melatonin suppresses estrogen function [61] and suppresses 5HT_2A _receptor activity [124]. Further, melatonin might be the link between E2 and helper T-cell (T_H_1) activity, as melatonin has been shown to upregulate expression of T_H_1-stimulating factors such as TNF-α and IFN-γ [125]. TNF-α increases the expression of MHC class II proteins and activates T_H_1 cells, [126] which are hallmarks of MS.

Similar MHC class II polymorphisms and T cell dysfunctions have been implicated in lupus,[127,128] and lower levels of free tryptophan[129] and MHC II protein over expression is also linked to autoimmune attack on beta cells in Type I diabetes (IDDM) [130]. Over expression of the MHC II following failure to select against self-reactive T-cells is also a useful model for rheumatoid arthritis, Graves disease, and Hashimoto's thyroiditis, in which T-cells react to proteins produced in the body, failing to discriminate them from invading organisms [131]. Women in whom estrogen-regulated serotonin signaling is compromised would be expected to have higher levels of MHC class II protein expression and may present these pathologies. However, simply over-expressing MHC II proteins is not sufficient to activate the immune system and induce autoimmune disorders [131]. The links between autoimmune disorders, serotonergic systems, and E2 suggest that manipulation of serotonin or E2 could be used to successfully treat these pathologies. Consistent with this suggestion, ER agonists reduce the symptoms of autoimmune disorders [132,133].

### Breast cancer

Carcinogenesis is conceptualized as consisting of three distinct phases: initiation, promotion and progression. Initiation is the irreversible alteration of a normal cell; promotion involves both proliferation of initiated cells and suppression of apoptosis of these cells; and progression is the irreversible conversion of one of the promoted initiated cells to an invasive, metastatic tumor cell [134]. Therefore, any endogenous milieu that induces apoptosis or suppresses mitogenesis of initiated cells could reduce breast cancer risk.

For breast cancer, one of the prevailing theories for the role of E2 is that longer duration of lifetime exposure to E2 is associated with increased risk, so that early menarche and late menopause result in greater likelihood of developing breast cancer [135]. Adding a role for serotonin does not conflict with this idea, but it does help explain several epidemiological findings that are not accounted for by a relationship between increased E2 exposure alone and breast cancer. First, the highest breast cancer incidence is in post-menopausal women, when endogenous E2 levels are much lower than before menopause. As described above, the higher E2 concentrations in the presence of progesterone prior to menopause cause an increase in 5HT_2A _receptor density and serotonin activity that promotes apoptosis. In contrast, 5HT_1A _activation (which occurs preferentially after menopause) decreases apoptotic signaling via caspase-3 suppression [38]. Therefore, if E2 is acting on breast cancer in part by serotonin modulation, then we would predict that the decrease in E2 after menopause should increase risk of breast cancer. This is consistent with the observed breast cancer incidence curve [136]. The failure of low levels of E2 to inhibit cancer growth is also reflected in patterns of tumor development within the estrous cycle. In mice, breast tumor growth occurs primarily in diestrus (when E2 is low), and tumor size is maintained or shrinks when E2 levels are high [137].

Second, in Pike's Breast Tissue Age model, a one-time rapid increase in breast tissue age and therefore breast cancer risk is included immediately following the first full-term pregnancy [138]. The extension of Pike's model includes multiple births by incorporating smaller increases in risk at each additional full-term pregnancy [139]. This pattern of increased risk for breast cancer immediately following full-term pregnancies is well-documented [140-142]. E2 concentrations increase steadily during pregnancy, peaking at about 100 times normal cycling levels [3]. In the days around parturition, these concentrations drop precipitously to levels below those of normal cycling females, where they are maintained for at least a month and potentially much longer (depending on suckling suppression) [143]. We postulate that the observed increase in breast cancer risk may be accounted for by the concurrent decrease in E2 and therefore changes in 5HT_2A _receptor function immediately prior to parturition. While E2's effect on serotonin could account for the immediate increase in risk, it cannot explain the long-term reduction in risk, which is likely related to other changes associated with parturition or lactation.

Third, obesity exerts differential effects on breast cancer risk over the lifespan; decreasing risk prior to menopause and increasing risk following menopause [144,145]. Under the prevailing theory of cumulative E2 exposure, obesity (which increases E2 levels[146]) would always be expected to increase breast cancer risk. However, the effect of E2 using serotonin mediation described above can account for the observed differential effects. Increased E2 in the presence of progesterone increases activation of 5HT_2A _receptors, while increased E2 in the absence of progesterone increases activation of 5HT_1A _receptors. The effects of these two receptors on apoptotic activity would predict that obesity exerts a protective effect before menopause and increases risk after menopause.

The importance of the presence of progesterone for this protective effect is underscored by recent HRT studies, which show that the use of estrogen and progesterone does not increase breast cancer risk,[147] while the use of estrogen and medroxyprogesterone (which decreases serotonin in some tissues[14,148]) has been shown to increase breast cancer risk. Consistent with the observed effects of HRT, oral contraception with Depo-Provera,^® ^which includes medroxyprogesterone rather than progesterone, has been shown to increase breast cancer risk [147, 149].

## Summary

Most research on pathologies in women's health has centered on changes in E2. Our review of data from a variety of fields suggests that serotonin is one way that estrogen is exerting its effects on physiology and pathology in women. The primary function of E2 is reproductive, and serotonergic mediation of the estrogen system likely provides reproductive benefits that are not yet understood. Several of the effects we have discussed could produce reproductive benefits: immune suppression during pregnancy could decrease the chance of lost pregnancies, postpartum activation of the immune system could increase antibodies in milk, increased clotting and vasoconstriction in the uterus could prevent bleeding during birth, and increased available calcium during lactation could improve the quality of breast milk. Notably, the same mechanism that results in these potential benefits in the reproductive system also produces changes in the remainder of the body that have consequences for women's physiology and pathologies. Whether the potential reproductive benefit of these effects is adequate to account for the maintenance of the estrogen/serotonin link remains to be explored. We suggest serotonergic mediation might contribute to explaining E2's effects on some pathologies, including heart attacks, multiple sclerosis, and breast cancer. Altering specific aspects of the serotonergic system, rather than simply increasing E2, could allow clinicians to target treatments in particular tissues or towards particular receptor types, alleviating undesirable side effects of E2 administration. Further studies are needed in order to unmask the precise molecular relationship between estrogen and serotonin and to document the clinical applications of this putative relationship.

## Abbreviations

CNS, central nervous system; E2, 17β-estradiol; 5HT, serotonin; TPH, tryptophan hydroxylase; SERT, serotonin reuptake transporter; ER β, estrogen receptor beta; ER α estrogen receptor alpha; NFKB, Nuclear Factor κB; PKC, Protein kinase C; cAMP, cyclic adenosine monophosphate; ERK, extra-cellular response kinase; HRT, hormone replacement therapy; T_H_1, helper T-cells type 1; T_H_2, helper T-cells type 2; MS multiple sclerosis; TNFα, Tumor necrosis factor α; IFNγ, Interferon γ; IDDM, insulin dependant diabetes mellitus.

## Competing interests

The author(s) declare that they have no competing interests.

## Authors' contributions

LAR developed the original idea and was primarily responsible for the content in the paper. MJB was responsible for verifying the effects of E2 in all systems and integrating the contents of the paper provided by other coauthors. DRP wrote and provided content in relation to breast cancer and epidemiologic review of the other pathologies as well as contributed to the writing of the rest of the manuscript. SMM provided cross species analysis and contributed to the writing of the manuscript. RMG wrote and provided content for the skeletal section. DLH contributed to the genetic information in this paper.

## Pre-publication history

The pre-publication history for this paper can be accessed here:


